# Continuous Exposure of Nonobese Adult Male Rats to a Soft-Textured, Readily Absorbable Diet Induces Insulin Resistance and Derangements in Hepatic Glucose and Lipid Metabolism

**DOI:** 10.1016/j.tjnut.2025.03.009

**Published:** 2025-03-10

**Authors:** Fumitake Yamaguchi, Sayaka Akieda-Asai, Eriko Nakamura, Hinano Uchida, Atsushi Yamashita, Yukari Date

**Affiliations:** 1 Frontier Science Research Center, University of Miyazaki, Miyazaki, Japan; 2 School of Nursing, Faculty of Medicine, University of Miyazaki, Miyazaki, Japan; 3 Department of Pathology, Faculty of Medicine, University of Miyazaki, Miyazaki, Japan

**Keywords:** eating habit, food texture, insulin resistance, lipid metabolism, type 2 diabetes

## Abstract

**Background:**

Type 2 diabetes (T2D) is characterized by insulin resistance and defective insulin secretion. Previously, we found that rats fed soft pellets (SPs) on a 3-h restricted schedule over 14 wk demonstrated glucose intolerance and insulin resistance with disruption of insulin signaling.

**Objectives:**

This study aims to determine *1*) the time required for an SP diet to induce insulin resistance, and *2*) whether the metabolic derangements in rats fed SPs can be reversed by changing to a standard control diet.

**Methods:**

We performed glucose tolerance tests and calculated the homeostasis model assessment of insulin resistance (HOMA-IR) to evaluate the insulinemic response to glucose and assess insulin resistance in nonobese male rats fed control pellets (CPs) or SPs on a 3–h restricted schedule (10:00–13:00) for 4 and 9 wk. At 11 wk, we switched half of the insulin-resistant SP group to CPs [soft-to-control pellets (SCPs)] and after an additional 11 wk evaluated changes in glucose and lipid metabolism across the 3 groups.

**Results:**

The glucose tolerance test results in the SP and CP rats did not differ at 4 or 9 wk. The insulin levels in the SP group were higher than in the CP group at both time points (*P* < 0.05). The HOMA-IR was significantly higher in the SP rats at 9 wk compared with the controls (*P* < 0.05). At 22 wk, the HOMA-IR, blood glucose levels at 30 min after initiating feeding, hepatic glucose metabolism, and lipid synthesis in rats fed SPs continuously were significantly greater than in those fed CPs (*P* < 0.05); however, these values in the SCP rats did not differ from those in the CP rats.

**Conclusions:**

A continuous diet of soft-textured, readily absorbable food may be an important and reversible underlying driver in T2D pathogenesis.

## Introduction

Type 2 diabetes (T2D) is one of the most common lifestyle-related diseases, with a worldwide prevalence of well over 500 million cases in 2021 [1] and is characterized by insulin resistance and ß-cell dysfunction. Diabetes is traditionally classified into type 1, characterized by pancreatic β-cell autoimmunity, and type 2 (T2D), characterized by β-cell dysfunction. In general, T2D has been associated with excessive nutritional intake that results in elevated insulin secretion, insulin resistance, and obesity [[2], [3], [4]]. However, a cohort study conducted in northern European countries [5] showed that 20% of patients with diabetes were nonobese, with a BMI < 25. Moreover, a large fraction of the Asian population with T2D is known to be nonobese [[6], [7], [8], [9]]. Indeed, Kashima et al. [10] showed that over 60% of their Japanese study participants with diabetes were not obese. Studies indicate that patients without obesity with diabetes have impaired insulin secretion or decreased insulin sensitivity compared with patients with obesity [[11], [12], [13]]; however, the precise lifestyle and molecular mechanisms that cause diabetes in patients without obesity remain to be fully elucidated.

We previously investigated the possible relationship between food texture and T2D onset by examining calorie intake, energy expenditure, and glucose and lipid metabolism in rats fed standard solid food pellets containing 51% carbohydrate, 25% protein, and 4.6% fat [control pellets (CPs)], or standard pellets with added water [soft pellets (SPs)], on a 3-h restricted feeding schedule [14,15]. Surprisingly, although there were no significant differences in calorie intake or body weight, the rats fed SPs showed glucose intolerance and insulin resistance. In addition, acetyl-CoA carboxylase (ACC) phosphorylation significantly decreased whereas fatty acid synthase (FASN) levels and triacylglycerol content significantly increased in the livers of rats fed SPs compared with rats fed CPs [15], indicating that the absorbable food texture of the SPs did not induce obesity but induced a rat equivalent of T2D (rT2D).

In the study we describe here, we first investigated the time it took for SP rats to develop glucose intolerance and insulin resistance by performing glucose tolerance tests (GTTs) at 4 wk and 9 wk in rats fed CPs or SPs. In addition, we assessed the presence of glucose intolerance and the HOMA-IR using blood glucose and plasma insulin levels before feeding. To investigate whether changing an SP diet to a CP diet reversed hepatic glucose and lipid metabolic dysfunction, we divided the SP rat group (*n* = 10) into an SP group (*n* = 5) and a soft-to-control pellet (SCP) group (*n* = 5) in which rats were fed SPs for 11 wk and CPs for the subsequent 11 wk. We compared the blood glucose, plasma insulin, and HOMA-IR levels before feeding. In addition, we monitored glucose and insulin levels of the 3 groups at pre-, mid-, and post-feeding, and examined levels of glucokinase (GCK) and glucokinase regulatory protein (GKRP) in their livers. Furthermore, we investigated metabolic alterations in the liver induced by SPs by analyzing the glycolysis-associated and gluconeogenesis-associated metabolites glucose 6-phosphate (G-6-P), fructose 6-phosphate (F-6-P), and pyruvic acid, using capillary electrophoresis time-of-flight mass spectrometry (CE-TOFMS) for cation analysis and CE-tandem mass spectrometry (CE-MS/MS) for anion analysis [16,17]. We also examined hepatic glucose 6-phosphatase catalytic subunit 1 (*G6Pc*) mRNA expression in the CP, SP, and SCP groups. To assess lipid metabolism, we evaluated hepatic ACC phosphorylation and *FASN* mRNA expression in the 3 groups. Finally, we investigated hepatic lipid accumulation histologically using Oil Red O staining and immunohistochemistry for adipophilin, a lipid component protein.

## Methods

### Animals

Fifteen 7-wk-old male Wistar rats (Charles River Japan) were individually housed in plastic cages maintained at constant room temperature under a 12-h light/12-h dark cycle (light: 08:00−20:00). The sample size was determined based on the findings of a previous study [15]. The rats were randomly divided into 2 groups (CP group, *n* = 5; SP group, *n* = 10) and fed either CPs or SPs for 11 wk. The CP group was fed standard laboratory feed pellets (4.8% fat, 49.5% carbohydrate, and 24.9% protein; CLEA Japan). The SP group was fed a mixture of standard laboratory feed pellets and water (standard laboratory feed pellets, 1 g; water, 1.4 mL). The nutrient composition of diets is shown in Table 1. The hardness of the CPs exceeded 200 N; the SP hardness was 0.32 N. Prepared SPs were stored at 4 °C and used within 1 wk. Visible bacteria counts were analyzed by the standard agar or potato dextrose agar plating method (Japan Food Research Laboratories). As a result, no mold or yeast growth occurred in the SPs. We investigated the time it took for SPs to induce glucose intolerance and insulin resistance using the 5 CP rats and 5 randomly selected SP rats. After 11 wk, we divided the SP group into an SP (*n* = 5) and an SCP group (*n* = 5) whose food was changed from SPs to CPs. The CP, SP, and SCP groups (*n* = 5 per group) were followed for an additional 11 wk, for a total observation period of 22 wk from the start of the experiments. All rats were fed between 10:00 and 13:00 on a 3-h restricted feeding schedule and were allowed ad libitum access to water throughout the experiments. Body weight and calorie intake were monitored weekly. All procedures were performed in accordance with the Japanese Physiological Society’s guidelines for animal care. The study protocol was approved by the University of Miyazaki Faculty of Medicine Ethics Review Committee for Animal Experimentation.

**TABLE 1:** Detailed composition of standard laboratory feed pellets.

Component	Content in 100 g of material
General nutrition facts and calories	
Moisture (%)	9.12
Crude protein (%)	24.91
Crude fat (%)	4.80
Crude fiber (%)	4.55
Crude ash (%)	7.13
Nitrogen-free extracts (%)	49.50
Energy (kcal)	340.8
Mineral nutrition facts	
Calcium (g)	1.15
Phosphorus (g)	1.11
Magnesium (g)	0.34
Potassium (g)	1.04
Manganese (mg)	12.10
Iron (mg)	33.81
Copper (mg)	1.13
Zinc (mg)	6.71
Sodium (g)	0.30
Vitamins	
Vitamin A (IU)	3150.0
Vitamin B_1_ (mg)	1.8
Vitamin B_2_ (mg)	1.4
Vitamin B_6_ (mg)	1.3
Total vitamin C (mg)	25.0
Vitamin D_3_ (IU)	255.0
Vitamin E (mg)	7.3
Pantothenic acid (mg)	3.1
Niacin (mg)	19.8
Folic acid (mg)	0.3
Choline (g)	0.2
Biotin (μg)	50.8
Inositol (mg)	709.0

### GTT and HOMA-IR of CP and SP rats at 4 and 9 wk

A GTT was performed on rats fed CPs or SPs for 4 wk or 9 wk (*n* = 5 per group). Rats were starved overnight and then injected intraperitoneally with glucose, 2 g/kg body weight [18], at 09:00. Glucose and plasma insulin levels from tail vein blood were measured immediately before injection (time point 0) and at 15, 30, 60, 120, and 180 min after injection. After measuring blood glucose using StatStrip XP3 (Nipro) [19], the remaining samples were collected in tubes containing a protease inhibitor cocktail (Roche), immediately centrifuged (2000 × *g* at 4 °C; 10 min), and stored at −80 °C until they were assayed. Plasma insulin levels were measured using ELISA kits (Morinaga). The AUC was calculated using Prism 8 (GraphPad Software).

HOMA-IR was determined using fasting insulin and blood glucose measurements according to the following formula [20,21]:

HOMA-IR = 26 × fasting insulin (ng/mL) × fasting glucose (mg/dL)/405.

### Computed tomography analysis of adipose tissues

At 4 and 9 wk, rats fed CPs or SPs (*n* = 5 per group) were anesthetized with pentobarbital (50 mg/kg intraperitoneally) and scanned by micro-computed tomography (CT; ALOKA LaTheta) at 1.5-mm intervals from the proximal end of L1 to the distal end of L6. The abdominal adiposity in each rat was determined using LaTheta software (version 1.00).

### Hepatic triacylglycerol content

At 4 and 9 wk, rats fed CPs or SPs (*n* = 5 per group) were allowed to starve overnight and were then deeply anesthetized with isoflurane inhalation (2.0 vol.%). To assay the hepatic triacylglycerol content, the lipids from 25 mg tissue were extracted in 1 mL of chloroform: methanol mixture [2:1 (vol:vol)] as previously described [15,20,22]. The triacylglycerol was quantified using a Triglyceride E test kit (FUJIFILM Wako Pure Chemical Corp.).

### Basal blood glucose, plasma insulin, HOMA-IR, glucose and insulin dynamics, and adipose tissue mass of CP, SP, and SCP rats

Blood samples were drawn from the tail vein of CP, SP, and SCP rats (*n* = 5 per group) starved overnight. Blood glucose and plasma insulin were measured as described previously. HOMA-IR of the rats in each group was calculated based on the values of glucose and insulin.

To investigate prandial and postprandial glucose and insulin levels, blood samples were drawn from the tail vein of CP, SP, and SCP rats before feeding (*T*_0_); at 30-, 60-, and 180-min intervals during feeding; and 30 min after feeding. Blood glucose and plasma insulin were measured as described previously. Adipose tissue mass of the CP, SP, and SCP rats was measured by CT as described in the Computed tomography analysis of adipose tissues section.

### Metabolite extraction

Approximately 20–40 mg frozen liver from the CP, SP, and SCP rats was placed in a homogenization tube, along with zirconia beads (5 mmϕ and 3 mmϕ). Next, 1200 μL of 50% acetonitrile/Milli-Q water containing internal standards [H3304-1002, Human Metabolome Technologies, Inc. (HMT)] was added to the tube, after which the tissue was completely homogenized at 1500 x g, 4ºC for 120 s using a beads shaker (Shake Master NEO, Bio Medical Science). The homogenate was then centrifuged at 2300 × *g*, 4ºC for 5 min. Subsequently, 800 μL of the upper aqueous layer was centrifugally filtered through a Millipore 5-kDa cutoff filter (Ultrafree-MC-PLHCC, HMT) at 9100 × *g*, 4ºC for 120 min to remove macromolecules. The filtrate was completely evaporated under vacuum and reconstituted in 50 μL of Milli-Q water for metabolome analysis at HMT.

### Metabolome analysis (*C-SCOPE*)

Metabolome analysis was conducted according to HMT’s *C-SCOPE* package, using CE-TOFMS for cation analysis and CE-MS/MS for anion analysis based on the methods described previously [16,17]. Briefly, CE-TOFMS and CE-MS/MS analyses were performed using an Agilent CE capillary electrophoresis system equipped with an Agilent 6210 time-of-flight mass spectrometer (Agilent Technologies, Inc.) and Agilent 6460 Triple Quadrupole LC/MS (Agilent Technologies), respectively. The systems were controlled by Agilent G2201AA ChemStation software version B.03.01 for CE (Agilent Technologies) and connected by a fused silica capillary (50 μm *i.d.* × 80 cm total length) with commercial electrophoresis buffer (H3301-1001 and I3302-1023 for cation and anion analyses, respectively, HMT) as the electrolyte. The time-of-flight mass spectrometer was scanned from *m/z* 50 to 1000 [16] and the triple quadrupole mass spectrometer was used to detect compounds in dynamic multiple mode. Peaks were extracted using MasterHands, automatic integration software (Keio University) [23], and MassHunter Quantitative Analysis B.04.00 (Agilent Technologies) to obtain peak information, including *m/z*, peak area, and migration time. Signal peaks were annotated according to HMT’s metabolite database based on their *m*/*z* values and migration times. The peak area of each metabolite was normalized to internal standards, and metabolite concentration was evaluated by standard curves with 3-point calibrations using each standard compound. Hierarchical cluster analysis and principal component analysis [24] were performed by HMT’s proprietary MATLAB and R programs, respectively. Detected metabolites were plotted on metabolic pathway maps using VANTED software [25].

### Western blotting

Twenty-two weeks after starting the experiments, the livers of the CP, SP, and SCP rats (*n* = 5 per group) were removed after overnight fasting. The samples (25 mg each) were homogenized in a mammalian cell lysis reagent (ProteoJET, Fermentas Life Sciences) containing a proteinase inhibitor cocktail (Roche Diagnostics) and phosphatase inhibitor cocktail (Roche Diagnostics). Thirty micrograms of total protein per sample were analyzed using SDS-PAGE (6%–10% acrylamide) and electroblotted onto polyvinylidene difluoride membranes (Immobilon-P; Millipore). The membranes were probed at 37°C for 1 h with gentle shaking with the primary antibody against GCK (1:2000; Proteintech Group, 19666-1-AP). Other membranes were then probed overnight at 4 °C with gentle shaking with primary antibodies against GKRP (1:1000; Cell Signaling Technology, #14328), sterol regulatory element binding protein 1 (SREBP1; 1:1000; Santa Cruz Biotechnology, sc-13551), carbohydrate response element binding protein (ChREBP; 1:1000; Novus Biologicals, NB400-135), ACC (1:1000; Cell Signaling Technology, #3662) and phospho-ACC (1:1000; Ser79; Cell Signaling Technology, #3661). All membranes were incubated for 1 h at room temperature with a horseradish peroxidase-labeled goat anti-rabbit IgG (H+L) antibody (1:10,000; Epitomics, #3053-1). Specific proteins were detected with an enhanced chemiluminescence system (Bio-Rad) in accordance with the manufacturer’s instructions. Western blotting for ACC was quantified by densitometry relative to β-actin by using the NIH image processing software, ImageJ (NIH).

### Quantitative PCR

To investigate the expression of hepatic *FASN* mRNA, and *G6Pc* mRNA, the livers of rats from each group (*n* = 5 per group) were removed and a total RNA from each tissue was rapidly extracted with Sepasol-RNA I Super G solution (Nacalai Tesque). First-strand cDNA was synthesized from total RNA by using a commercially available kit (PrimeScript RT reagent kit; Takara Bio). The resulting samples were subjected to quantitative PCR. Quantitative PCR assays were conducted on a QuantStudio 5 Real-Time PCR System (Applied Biosystems) by using SYBR Premix Ex Taq II reagents (Takara Bio). The relative abundance of the reaction products was normalized to the level of *hypoxanthine phosphoribosyltransferase 1* mRNA, which was unaffected by experimental factors, and calculated using the 2(-Delta Delta C(T)) Method [26]. The primer sets for all of these genes are listed in Table 2.

**TABLE 2:** Primers used for quantitative PCR analysis.

Gene	Accession no	Product size (bp)	Forward (5′ → 3′)	Reverse (5′ → 3′)
G6Pc	NM_013098.2	62	CTCACTTTCCCCATCAGGTG	GAAAGTTTCAGCCACAGCAA
FASN	NM_017332	126	AGTTCTGGGCCAACCTCATTG	AGGCGTCGAACTTGGACAGAT
HPRT	NM_012583	81	TCATGAAGGAGATGGGAGGC	CCAGCAGGTCAGCAAAGAACT

Abbreviations: G6Pc, glucose-6-phosphatase catalytic subunit 1; FASN, fatty acid synthase; HPRT, hypoxanthine phosphoribosyltransferase 1.

### Hepatic triacylglycerol content of CP, SP, and SCP rats

CP, SP, and SCP rats (*n* = 5 per group) were allowed to fast overnight and were then deeply anesthetized with isoflurane inhalation (2.0 vol.%). The hepatic triacylglycerol contents of the 3 groups were assayed as described previously.

### Histological analysis

Twenty-two weeks after starting the experiments, livers from the 3 groups were rapidly frozen in liquid nitrogen and stored without fixation [27]. The frozen samples were then subjected to Oil Red O staining with a Lipid Assay Kit (Primary Cell Co., Ltd.). The frozen liver samples were cut into 10-μm-thick sections and kept at −30°C. Briefly, the frozen liver sections were washed with phosphate-buffered saline (PBS) and stained in fresh Oil Red O solution [Oil Red O stock solution; H_2_O, 3:2 (vol:vol)]. The stain was removed, and the sections were washed 3 times with water and then photographed. All images of the Oil Red O-stained sections were obtained with an All-in-One Fluorescence Microscope (BZ-X800, KEYENCE) using a Plan Apochromat 40× objective (NA0.95, BZ-PA40, KEYENCE). Oil Red O-positive areas and signaling intensities were automatically calculated using a hybrid cell count application (BZ-H4C, KEYENCE) in the BZ-X Analyzer software (BZ-H4A, KEYENCE) [28], by an examiner who was blinded to the experimental group information.

To examine immunoreactivity of adipophilin, livers from CP, SP, and SCP rats (*n* = 5 per group) were fixed for 24 h with 3.7% formaldehyde at 4°C, dehydrated, embedded in paraffin, and cut into 3-μm-thick sections. The sections were depleted of paraffin using xylene, then subjected to immunohistochemistry. After microwave treatment (95°C, 20 min, citrate buffer pH 6), the sections were treated with 0.3% hydrogen peroxide for 15 min to inactivate endogenous peroxidases and 5% skim milk in PBS (pH 7.4) for 3 h, and then incubated overnight at 4°C with an anti-adipophilin antibody (OriGene Technologies). After the sections were washed with PBS for 15 min, they were incubated for 30 min with peroxidase-labeled polymer conjugated to goat anti-mouse immunoglobulins (EnVision+ System, Dako/Agilent). The horseradish peroxidase activity was visualized using 3,3′-diaminobenzidine solution containing hydrogen peroxide, and the sections were counterstained with Mayer’s hematoxylin. The adipophilin immunopositive areas were semiquantified using WinROOF color image analysis software (Mitani Corp.) [29]. The microscopic digital images captured the 5 highest density fields under a 40× objective lens in each slide. The immunopositive areas were extracted as green areas using specific protocols based on the color parameters of hue, lightness, and saturation. Because hepatic stellate cells express adipophilin [30], we deleted the immunopositive ones with an image analysis software eraser tool before the extraction (Supplemental Figure 1).

### Statistical analysis

Data were analyzed using the Statistical Package for the Prism GraphPad software version 9.5.1 for Windows. All data are presented as mean ± SEM. Statistical significance was evaluated using Student’s *t*-test, Mann-Whitney *U* tests, and 1-way or 2-way analysis of variance. Fisher’s protected least significant difference was used as a post hoc test to compare CP differences. *P* values <0.05 were considered significant (2-tailed tests).

## Results

### Calorie intake and body weight of rats fed CPs or SPs for 11 wk

**FIGURE 1:**
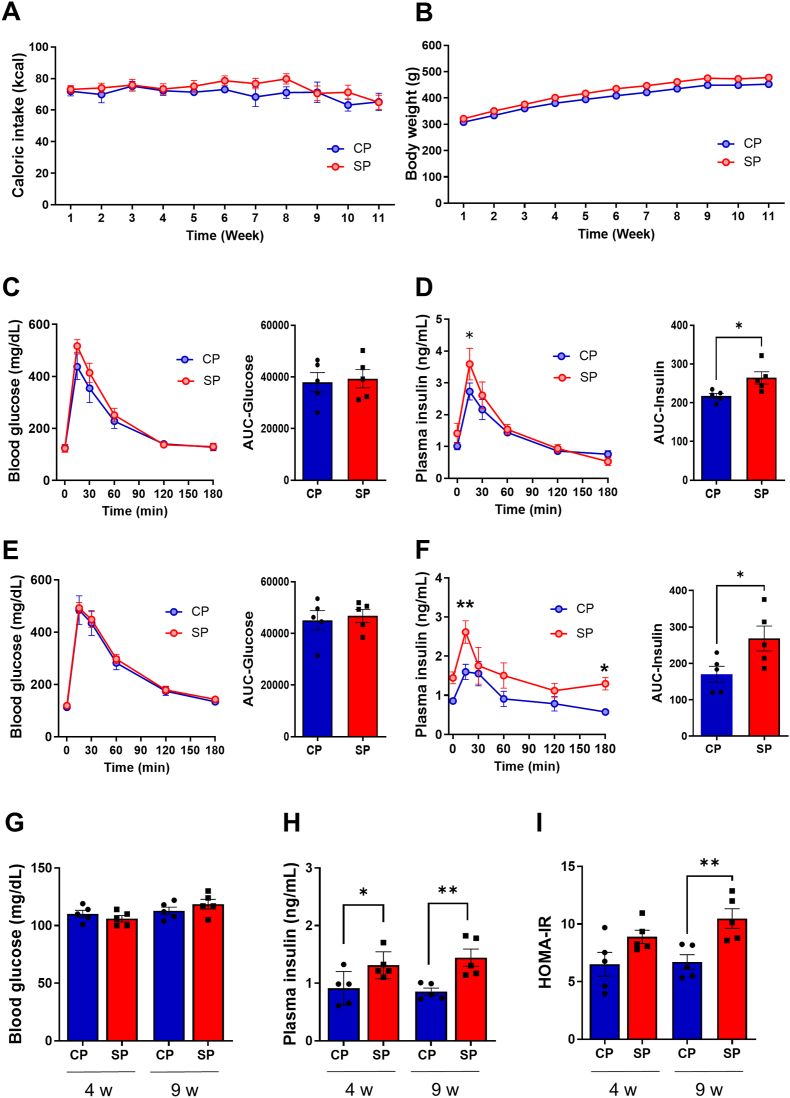
Caloric intake, body weight, and glucose metabolism of rats fed soft pellets (SP) or control pellets (CP) for 4 wk and 9 wk. (A) Caloric intake of rats fed CPs or SPs for 9 wk. (B) Body weight of rats fed CPs or SPs for 9 wk. (C–F) Blood glucose and insulin levels as determined by glucose tolerance testing (GTT): Blood glucose levels of rats fed SPs or CPs for 4 wk (C) and 9 wk (E). Plasma insulin levels of rats fed SPs or CPs for 4 wk (D) and 9 wk (F). (G) Blood glucose levels with overnight fasting of rats fed SPs or CPs for 4 wk and 9 wk. (H) Plasma insulin levels with overnight fasting of rats fed SPs or CPs for 4 wk and 9 wk. (I) Overnight-fasting HOMA-IR levels in rats fed SPs or CPs for 4 wk and 9 wk. *n* = 5 per group. ∗*P* < 0.05; ∗∗*P* < 0.01 compared with rats fed CPs.

Throughout the experimental period, all animals were included in the analysis. To investigate the influence of food texture, we monitored the calorie intake and body weight of rats fed either CPs or SPs for 11 wk. As in our previous studies, there were no significant differences in calorie intake (*P* = 0.237) and body weight (*P* = 0.118) between the CP and SP groups (Figure 1A and B) [14,15,20].

### GTT and HOMA-IR of CP and SP rats at 4 and 9 wk

The GTT showed no significant differences in blood glucose levels between the CP and SP groups at 4 wk (*P* = 0.112) or 9 wk (*P* = 0.536) after starting the experiments (Figure 1C and E). In contrast, insulin levels 15 min after glucose injection were significantly higher in the SP group than in the CP group at 4 wk (*P* = 0.023) and 9 wk (*P* = 0.005) (Figure 1D and F). Although the AUCs for the glucose responses of the CP and SP groups did not differ at either 4 wk (*P* = 0.795) or 9 wk (*P* = 0.716) (Figure 1C and E), the AUCs for the insulin responses in the SP group at 4 wk or 9 wk were higher than in the CP group (4 wk, *P* = 0.025; 9 wk, *P* = 0.042) (Figure 1D and F). There were no significant differences in blood glucose after overnight fasting between the CP and the SP groups at either 4 (*P* = 0.337) or 9 wk (*P* = 0.306), whereas fasting plasma insulin levels in the SP group were notably higher than in the CP group at 4 wk (*P* = 0.043) and 9 wk (*P* = 0.006) (Figure 1G and H). The HOMA-IR at 9 wk was significantly higher in the SP group than in the CP group (*P* = 0.008) (Figure 1I).

### Adipose tissue mass and hepatic triacylglycerol content of CP and SP rats at 4 and 9 wk

**FIGURE 2:**
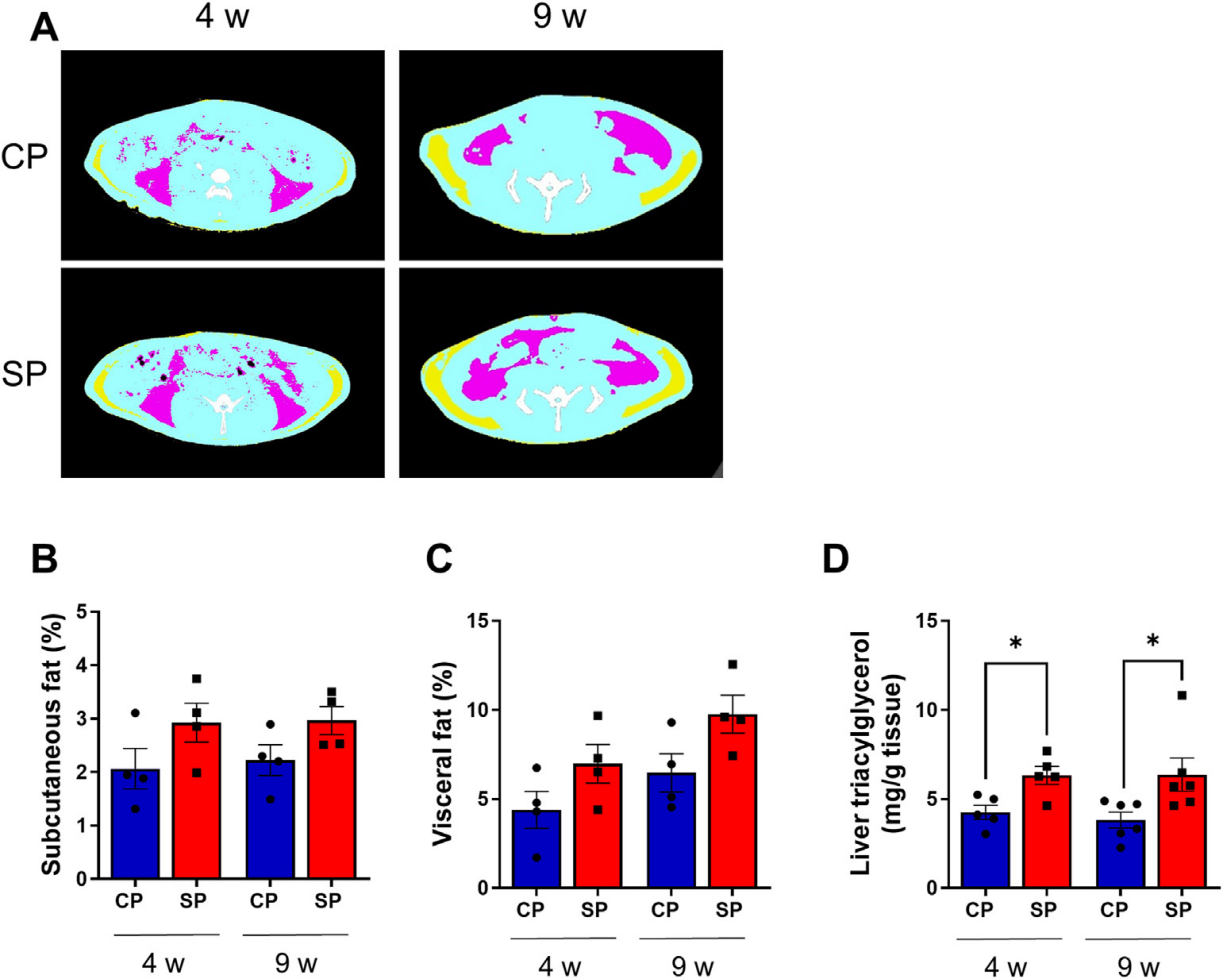
Adipose tissue mass and hepatic triacylglycerol content of CP and SP rats at 4 and 9 wk. (A) Axial computed tomography (CT) images of rats fed SPs or CPs, indicating areas of subcutaneous (yellow) and visceral (pink) fat and muscle (blue). (B, C) CT-derived percentages of subcutaneous and visceral fat (axial view, proximal L1 to distal L6). *n*  =  4 per group. (D) Hepatic triacylglycerol content in rats fed SPs or CPs for 4 wk and 9 wk. *n* = 5 per group. ∗*P* < 0.05 compared with rats fed CPs. CP, control pellets; SP, soft pellets.

Subcutaneous and visceral fat tissue were identified on CT slices using internal CT software (Figure 2A). There were no significant differences in the percentages of subcutaneous and visceral fat between CP and SP rats at either 4 wk (subcutaneous fat tissue, *P* = 0.151; visceral fat tissue, *P* = 0.136) or 9 wk (subcutaneous fat tissue, *P* = 0.104; visceral fat tissue, *P* = 0.072) (Figure 2 B and C). In contrast, the SP group had more hepatic triacylglycerol than the CP group at 4 wk (*P* = 0.012) and 9 wk (*P* = 0.033) (Figure 2D).

### Calorie intake and body weight of CP, SP, and SCP rats from weeks 12 to 22

**FIGURE 3:**
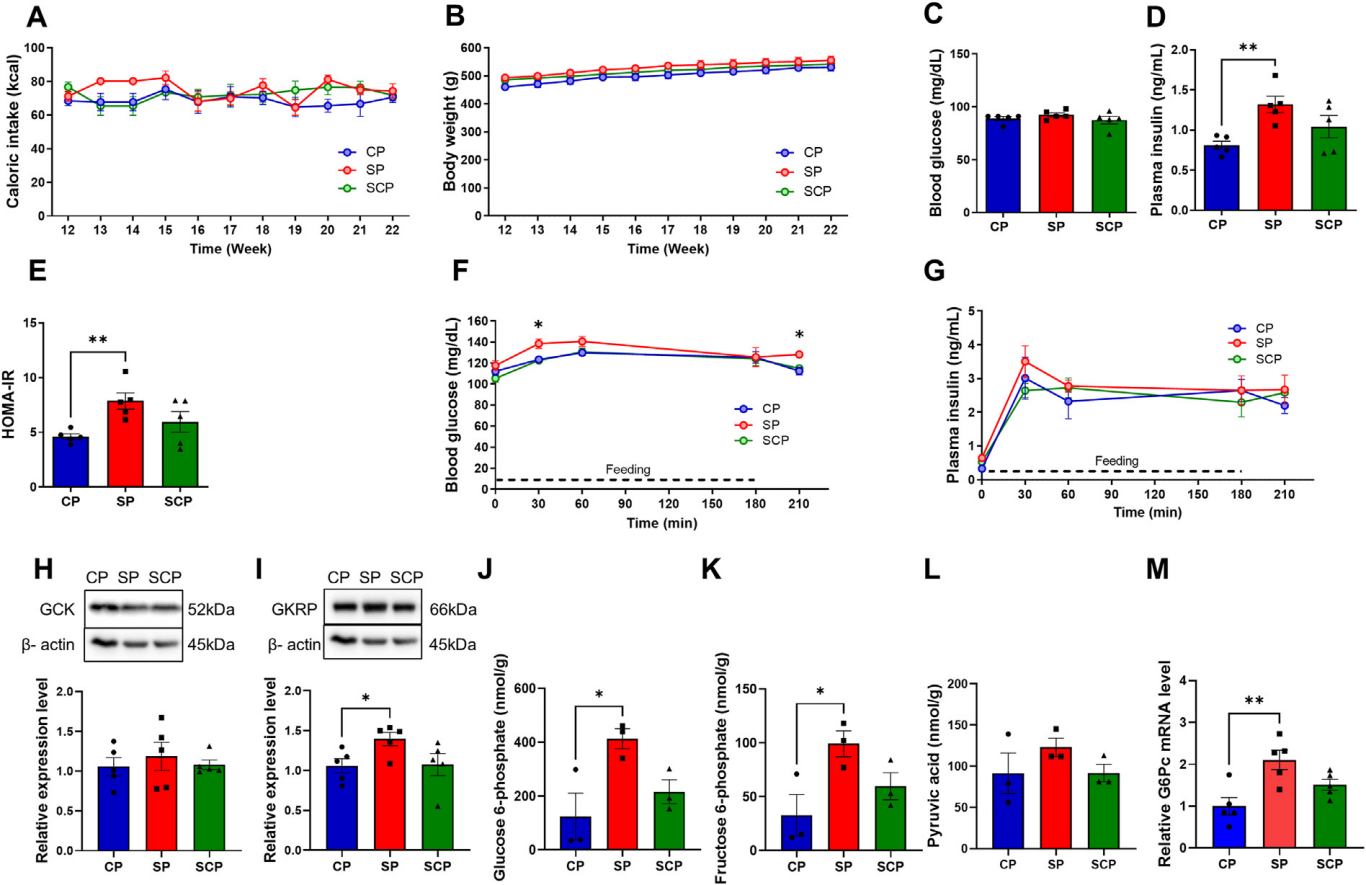
Effects of dietary changes on caloric intake, body weight, and glucose metabolism. (A) Caloric intake and (B) body weight in rats fed CPs or SPs for 22 wk, and rats fed SPs for 11 wk before switching to CPs for 11 wk (SCP group). (C) Overnight-fasting blood glucose levels, (D) overnight-fasting plasma insulin levels, and (E) HOMA-IR in CP, SP, and SCP rats. (F) Prandial and postprandial blood glucose levels and (G) plasma insulin levels in CP, SP, and SCP rats. (H, I) Representative blots and expression levels of (H) hepatic glucokinase (GCK) and (I) glucokinase regulatory protein (GKRP) in CP, SP, and SCP rats (*n* = 5 per group). These bands were cropped from the full blot images shown in Supplementary Figure S2. Metabolome analysis (J–L): (J) glucose 6-phosphate (K) fructose 6-phosphate, and (L) pyruvic acid in the livers of CP, SP, and SCP rats (*n* = 3 per group). (M) Relative mRNA expression levels of glucose 6-phosphatase catalytic subunit 1 (G6Pc) in CP, SP, and SCP rats (*n* = 5 per group). ∗*P* < 0.05; ∗∗*P* < 0.01 compared with rats fed CPs. CP, control pellets; SCP, soft-to-control pellet; SP, soft pellets.

To examine whether changing SPs to CPs influences calorie intake and body weight, we divided the SP group into an SP and an SCP group. There were no significant differences in calorie intake (*P* = 0.272) or body weight (*P* = 0.266) between the 3 groups (Figure 3A and B).

### Basal blood glucose, plasma insulin, and HOMA-IR of CP, SP, and SCP rats

There were no significant differences in blood glucose levels between the CP, SP, and SCP groups with overnight fasting (Figure 3C) at 22 wk after starting the experiments (*P* = 0.407). In addition, whereas the fasting plasma insulin levels of the SP rats were markedly higher than those of the CP rats (*P* = 0.008), the insulin levels of the CP and SCP rats were not significantly different (*P* = 0.228) (Figure 3D). Similarly, the HOMA-IR levels were higher in the SP rats than in CP rats (*P* = 0.007), whereas the levels in the SCP and CP rats did not differ (*P* = 0.207) (Figure 3E).

### Prandial and postprandial blood glucose and plasma insulin levels of CP, SP, and SCP rats

The glucose and insulin responses to feeding are shown in Figure 3F and G. Blood glucose levels of the SP rats were higher than those of the CP rats at 30 min after initiating feeding (*P* = 0.028) and at 30 min after completing the meal (*P* = 0.011) (Figure 3F). There were no statistically significant differences in the insulin levels among the 3 groups (*P* = 0.260) (Figure 3G).

### GCK, GKRP, and G6Pc expression

The GCK expression was not significantly different among the 3 groups (*P* = 0.748) (Figure 3H, Supplemental Figure 2). Conversely, whereas the GKRP expression in the SP group was markedly higher than in the CP group (*P* = 0.045) (Figure 3I, Supplemental Figure 2), no significant difference was noted between the CP and SCP groups (*P* = 0.925) (Figure 3I, Supplemental Figure 2). The level of *G6Pc* mRNA, a rate-limiting gluconeogenesis enzyme, was higher in the SP group than in the CP group (*P* = 0.002) (Figure 3M). There was no significant difference in *G6Pc* mRNA expression between the SCP and CP groups (*P* = 0.086) (Figure 3M).

### Metabolic alterations in glycolysis and lipogenesis

We investigated alterations in glucose metabolism in the livers of CP, SP, and SCP rats. The levels of G-6-P and F-6-P upstream of pyruvic acid were significantly higher in the SP rats than in the CP rats (G-6-P, *P* = 0.026; F-6-P, *P* = 0.035) (Figure 3J and K), whereas no significant differences in these metabolite levels were found between the CP and SCP rats (G-6-P, *P* = 0.493; F-6-P, *P* = 0.392) (Figure 3J and K). There were no significant differences in the pyruvic acid levels between the 3 groups (*P* = 0.372) (Figure 3L).

### Lipogenesis factor expression and hepatic triacylglycerol content

**FIGURE 4:**
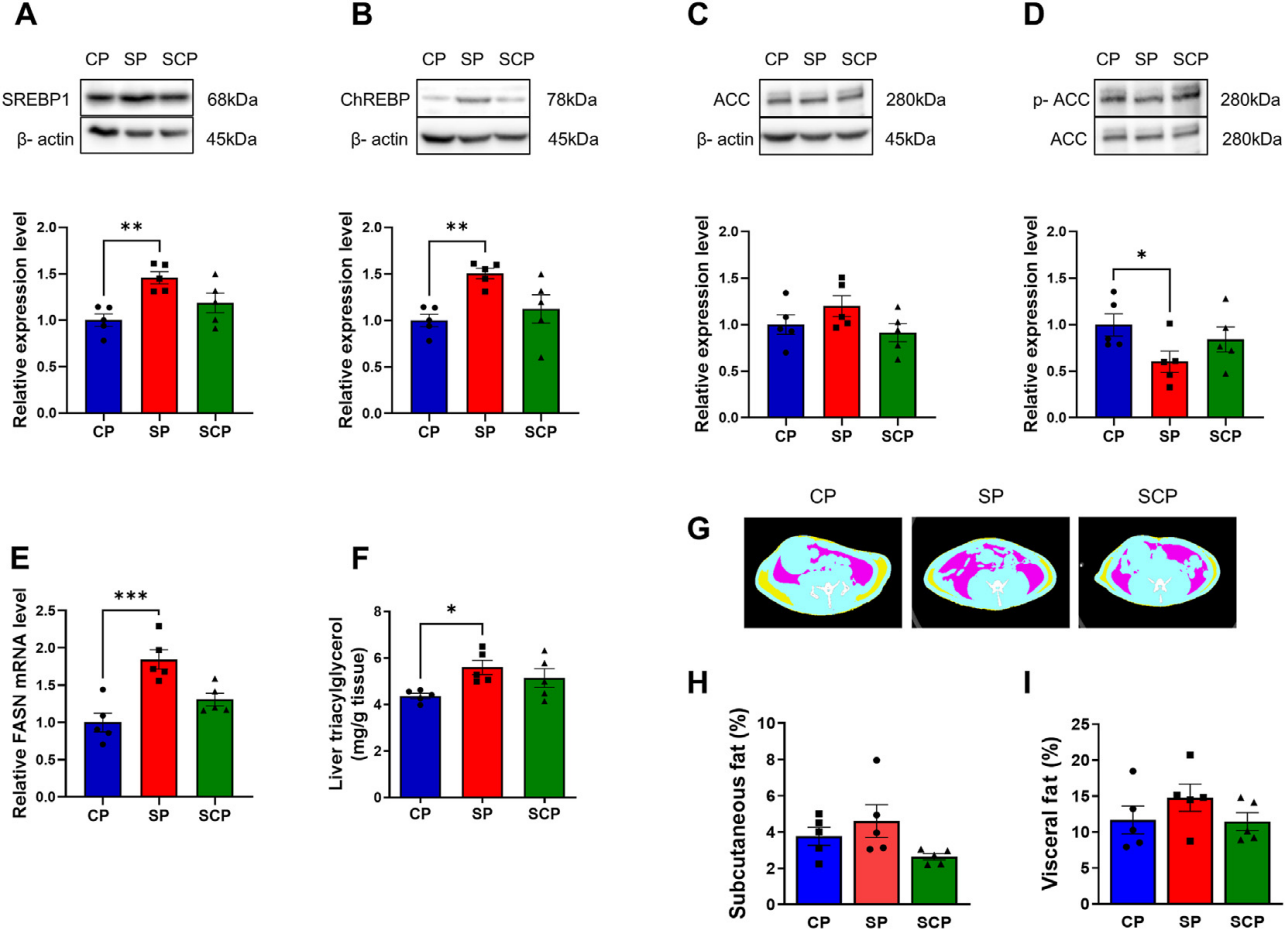
Lipid synthesis in the livers of rats fed CPs or SPs for 22 wk, and rats fed SPs for 11 wk before switching to CPs for 11 wk (SCP rats). (A–D) Representative blots and expression levels of (A) sterol regulatory element binding protein 1 (SREBP1), (B) carbohydrate response element binding protein (ChREBP), (C) hepatic acetyl-CoA carboxylase (ACC) and (D) phosphorylated ACC (p-ACC) in the CP, SP, and SCP rats. These bands were cropped from the full blot images shown in Supplementary Figure S2. (E) Relative fatty acid synthase (FASN) mRNA expression levels in CP, SP, and SCP rats. (F) Hepatic triacylglycerol content levels in the CP, SP, and SCP rats. (G) Axial computed tomography (CT) images of SP, CP, and SCP rats indicating areas of subcutaneous (yellow) and visceral (pink) fat and muscle (blue). (H, I) CT-derived percentages of subcutaneous and visceral fat (axial view; proximal L1 to distal L6). *n* = 5 per group. ∗*P* < 0.05; ∗∗*P* < 0.01; ∗∗∗*P* < 0.001 compared with rats fed CPs. CP, control pellets; SCP, soft-to-control pellet; SP, soft pellets.

To explore the relationship between diet texture and alterations in hepatic lipid metabolism, we investigated SREBP1, ChREBP, ACC expression and phosphorylation, *FASN* mRNA expression, and triacylglycerol content in the liver. SREBP1 and ChREBP expression in SP rats were significantly increased compared with those in CP rats (SREBP1, *P* = 0.002; ChREBP, *P* = 0.004), whereas the CP and SCP rats showed no significant difference in expression levels of these binding proteins (SREBP1, *P* = 0.133; ChREBP, *P* = 0.407) (Figure 4A and B, Supplemental Figure 2). There were no statistically significant differences in ACC expression between the 3 groups (*P* = 0.184) (Figure 4C, Supplemental Figure 2). Phosphorylation of ACC was significantly decreased in the SP group compared with the CP group (*P* = 0.042), whereas no significant differences between the CP and SCP groups were noted (*P* = 0.389) (Figure 3D, Supplemental Figure 2). In addition, the *FASN* mRNA levels in the SP group were higher than in the CP group (*P* < 0.001), whereas there was no significant difference between the CP and SCP groups (*P* = 0.148) (Figure 4E). Corresponding to the alterations in hepatic lipogenic factors in the SP group, the hepatic triacylglycerol contents in the SP group were significantly higher than in the CP group (*P* = 0.014), whereas no significant differences in the hepatic triacylglycerol contents were found between the CP and SCP groups (*P* = 0.093) (Figure 4F).

### Adipose tissue mass in the CP, SP, and SCP rats

Subcutaneous and visceral fat tissue were identified on CT slices using internal CT software (Figure 4G). There were no significant differences in the percentages of subcutaneous and visceral fat between the 3 groups (subcutaneous fat tissue, *P* = 0.109; visceral fat tissue, *P* = 0.347) (Figure 4H and I).

### Histological analysis

**FIGURE 5:**
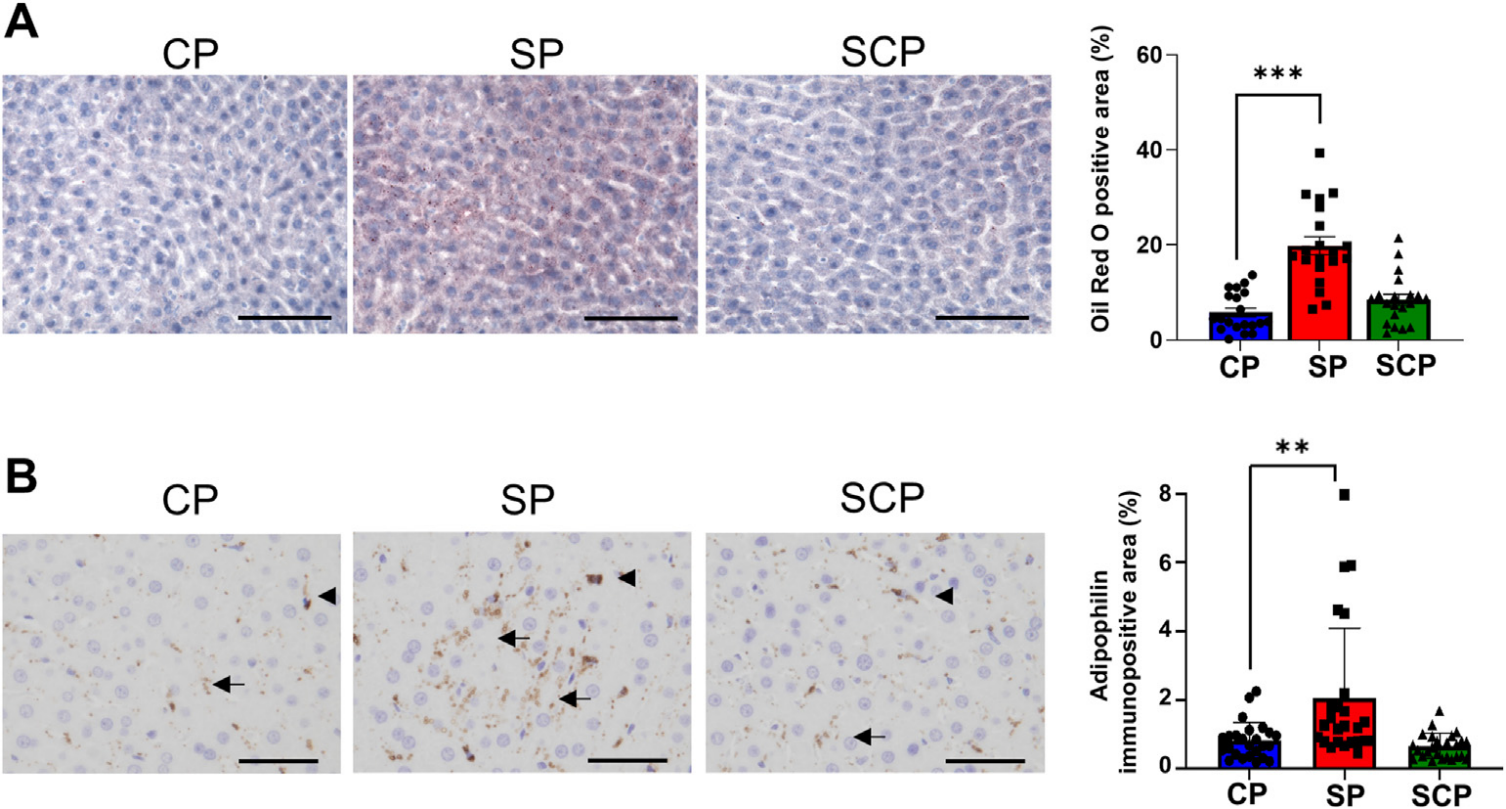
Hepatic lipid accumulation and adipophilin expression in rats fed CPs or SPs for 22 wk, and rats fed SPs for 11 wk before switching to CPs for 11 wk (SCP group). (A) Oil Red O staining in the livers of CP, SP, and SCP rats (*n* = 20 images per group). (B) Immunohistochemical images and adipophilin immunopositive areas in the livers of CP, SP, and SCP rats. Adipophilin expression in hepatocytes (arrows) and in hepatic sinusoidal cells (arrowheads) suggesting hepatic stellate cells (*n* = 25 images per group). ∗∗*P* < 0.01; ∗∗∗*P* < 0.001 compared with rats fed CPs. CP, control pellets; SCP, soft-to-control pellet; SP, soft pellets.

We performed Oil Red O staining to investigate lipid accumulation in the livers of the CP, SP, and SCP rats. Oil Red O staining demonstrated that hepatic lipid accumulation significantly increased in the SP rats compared with the CP rats (*P* < 0.001), whereas no significant differences in lipid accumulation were seen between the CP and SCP groups (*P* < 0.184) (Figure 5A). We also examined the expression of adipophilin, a lipid droplet protein, in the livers of CP, SP, and SCP rats. As shown in Figure 5B, adipophilin was expressed in hepatocytes and hepatic sinusoidal cells likely to be stellate cells. To measure the adipophilin immunopositive area in hepatocytes, we semiquantified the immunopositive area after deleting the immunopositive sinusoidal cells with image analysis software. The adipophilin immunopositive area in the SP group was significantly larger than in the CP group (*P* = 0.006) (Figure 5B). There were no significant differences in the adipophilin immunopositive areas between the CP and SCP groups (*P* > 0.999) (Figure 5B).

## Discussion

In this study, we investigated how long it took rats fed a soft diet for 4 and 9 wk to develop disordered glucose metabolism. Our data demonstrated that rats fed SPs for 4 wk on a 3-h-restricted schedule already showed insulin hypersecretion, whereas by 9 wk, rats fed SPs had developed insulin resistance without their GTTs showing a diabetic pattern. In addition, although the subcutaneous and visceral fat mass of rats fed SPs for 4 and 9 wk did not differ from those of rats fed CPs, the hepatic triacylglycerol in rats fed SPs was significantly greater than that of rats fed CPs for either 4 or 9 wk. Together, these findings suggest that habitual ingestion of SPs first induces insulin hypersecretion in response to glucose administration and subsequently induces insulin resistance. Moreover, SP rats may use fat converted from glucose in the liver as energy at a quite early time point. We have yet to elucidate how habitual SP ingestion triggers insulin resistance and a subsequent diabetic pattern in GTTs [14]. Further investigations into the absorption of SPs in the intestine, accelerated hypersecretion of insulin, and conversion of glucose to energy in the livers of SP rats are needed to clarify the molecular mechanisms by which SPs induce insulin resistance and glucose intolerance.

To investigate whether an SP diet is a factor in rT2D pathogenesis, we examined calorie intake, body weight, blood glucose, and plasma insulin in an additional (SCP) group comprised of rats fed SPs for 11 wk before changing to a CP diet for the remaining 11 wk of the study. Again, there were no significant differences in calorie intake, body weight, or fasting blood glucose levels between the 3 groups; however, plasma insulin and HOMA-IR levels in the SP group were markedly higher than in the CP group. On the other hand, plasma insulin levels and HOMA-IR in the SCP and CP groups did not differ, suggesting that changing an SP diet to a CP diet can reverse insulin resistance. We previously showed that either insulin receptor substrate 2 expression or protein kinase B phosphorylation after insulin injection, involved in insulin signaling, were decreased in the liver of SP rats [15]. In this study, we have yet to investigate whether an abnormality of insulin signaling in SP rats could be reversed by changing from a soft to a control diet. We plan to address these issues in conjunction with the mechanisms of insulin secretion induced by a soft diet in a future study.

In our previous study, we observed less food remaining in the stomach at 5 and 10 h after feeding in SP rats compared with CP rats [15], indicating that the nutrient load reaches the intestine faster, thus facilitating glucose absorption in the SP group relative to the CP group. On the basis of these observations, we investigated preprandial, prandial, and postprandial glucose and insulin levels in our CP, SP, and SCP rats. As predicted, the SP group’s glucose levels at 30 min into feeding and 30 min after the meal were higher than the CP group’s glucose levels, whereas no significant differences were noted between CP and SCP rats at any point.

To assess alterations in hepatic glucose metabolism, we examined the expression of GCK and GKRP. GCK catalyzes the first step in glucose metabolism (phosphorylation of glucose to G-6-P) and regulates the uptake and storage of dietary glucose [31,32]. We postulated that the continuous exposure of the SP rat hepatocyte to high glucose would accelerate hepatic glucose uptake. Alternatively, GKRP is known to regulate the activity and distribution of GCK in hepatocytes by forming an inhibitory complex with GCK [31,32]. In this study, although GCK expression in the SP rats was not significantly higher than in the CP or SCP rats, GKRP expression increased significantly in the SP rats compared with the CP rats. These data suggest that by binding GCK, the abundant GKRP may have inhibited GCK to restore a more physiological metabolic state. We also showed, using metabolome analysis, that G-6-P and F-6-P, 2 glycolysis metabolites, significantly increased in the SP group compared with the CP group. These data indicate that hepatic glycolysis could not keep pace with the increased blood glucose from accelerated absorption in the small intestines of SP rats. Furthermore, the increased expression of hepatic *G6Pc* mRNA indicates that gluconeogenesis could be enhanced in SP rats. *G6Pc* hydrolyzes G-6-P to glucose [33] and is a rate-limiting enzyme in the gluconeogenesis pathway [[34], [35], [36]]. Taken together, our findings suggest that the increase in G-6-P and F-6-P in the livers of SP rats may be caused by insufficient glycolysis and/or enhanced gluconeogenesis.

Insulin is a strong activator of the lipogenic pathway through the activation of the lipogenic transcription factors SREBP1 and ChREBP [37]. We previously showed that SREBP1 and ChREBP, known major mediators of hepatic insulin and glucose signaling, are significantly increased in the livers of SP rats compared with CP rats [15]. We also showed that ACC phosphorylation markedly decreased in SP rats compared with CP rats [15], and hepatic *FASN* mRNA expression and triacylglycerol content increased in SP rats more than in CP rats [15]. In the current study, we again found a significant increase in SREBP1, ChREBP, hepatic *FASN* mRNA expression, and triacylglycerol content and a significant decrease in ACC phosphorylation in the SP rats, indicating an acceleration of lipogenesis in their livers. Furthermore, the accelerated lipogenesis improved by changing from an SP diet to a CP diet. Corresponding to the biochemical data for lipogenesis, our histological studies showed that the increased hepatic Oil Red O-positive area and adipophilin immunoreactivity in the SP rats were reduced after changing from an SP diet to a CP diet, indicating that the dietary switch reversed the accumulation of lipids in the SP rat livers. The amelioration of lipid accumulation with switching from an SP to a CP diet suggests that glucose intolerance and/or insulin resistance induced by the habitual eating of SPs contributes to enhanced hepatic lipogenesis.

In summary, our findings suggest that a diet consisting of SPs on a 3-h-restricted schedule rapidly induces insulin hypersecretion and leads to the development of rT2D in the absence of hyperphagia and overweight. Recently, it was reported that light-phase time-restricted feeding significantly reduces insulin signaling compared with ad libitum feeding [38]. Therefore, it is possible that the 3-h-restricted schedule may have affected glucose and lipid metabolism in our study also. On the other hand, we clarified that rats fed SPs ad libitum also display disorders of lipid metabolism [39]. Thus, our data indicate that in addition to a 3-h-restricted feeding schedule, a diet consisting of soft-textured, readily absorbable food induces metabolic derangements in nonobese rats that mimic T2D. Moreover, we revealed that the glucose intolerance, insulin resistance, hepatic glucose metabolism, and lipid accumulation in SP rats are reversed by changing from a soft diet to a standard control diet. A diet of SP foods can be more easily absorbed, thereby inducing a persistent state of hyperglycemia and hyperinsulinemia. Because of the rapid absorption of an SP-based diet that induced hyperglycemia and hyperinsulinemia in our experimental rats, we speculate that the increased glucose and insulin transported to the liver resulted in the abnormalities in the glucose metabolism we observed. Together, these findings strongly indicate that the involvement of the digestive system in glucose metabolism may be an important factor in the onset of SP-induced T2D. Moreover, we predict that the expression of sodium-glucose cotransporter 1 (SGLT1) and the translocation of glucose-transporter 2 (GLUT2) in the small intestine play important roles in glucose absorption. In fact, we found that SGLT1 was markedly increased in the small intestines of SP rats (preliminary data not shown). Further research into the absorption rate of SPs, the change in SGLT1 expression during a meal, and the linkage of SGLT1 with GLUT2 in the small intestine is needed to fully elucidate the differences in glucose absorption between an SP diet and a control diet.

## Author contributions

The authors’ responsibilities were as follows – FY, EN, HU, SA-A: performed the animal experiments and molecular biological analyses; SA-A, AY: contributed to overseeing the experiments; YD: designed the experiments; FY, AY, YD: wrote the manuscript; and all authors: read and approved the final manuscript.

## Data availability

The datasets generated and/or analyzed during this study are available from the corresponding author on reasonable request.

## Funding

This study was supported in part by grants-in-aid for Scientific Research (nos. JP22592340, JP25560056 and JP21K21142) from the Ministry of Education, Culture, Sports, Science and Technology of Japan; Educational grant from Abbott Medical Japan LLC.

## Conflict of interest

FY reports financial support was provided by Abbott Japan Co Ltd. YD reports financial support was provided by Japan Society for the Promotion of Science. Other authors declare that they have no known competing financial interests or personal relationships that could have appeared to influence the work reported in this paper.
