# Supplementary material for: Continuous Exposure of Nonobese Adult Male Rats to a Soft-Textured, Readily Absorbable Diet Induces Insulin Resistance and Derangements in Hepatic Glucose and Lipid Metabolism
Source: J Nutr. 2025 Mar 10;155(5):1387–97. doi: 10.1016/j.tjnut.2025.03.009 (PMC12121407; doi:10.1016/j.tjnut.2025.03.009)
Supplement: multimedia component 1 [file mmc1.docx]

**Supplementary Materials**

**Continuous exposure of non-obese adult male rats to a soft-textured, readily absorbable diet induces insulin resistance and derangements in hepatic glucose and lipid metabolismt**

Fumitake Yamaguchi, Sayaka Akieda-Asai, Eriko Nakamura, Hinano Uchida,

Atsushi Yamashita, and Yukari Date

Corresponding author email: dateyuka@med.miyazaki-u.ac.jp

**This file includes:**

Supplementary Figures

**
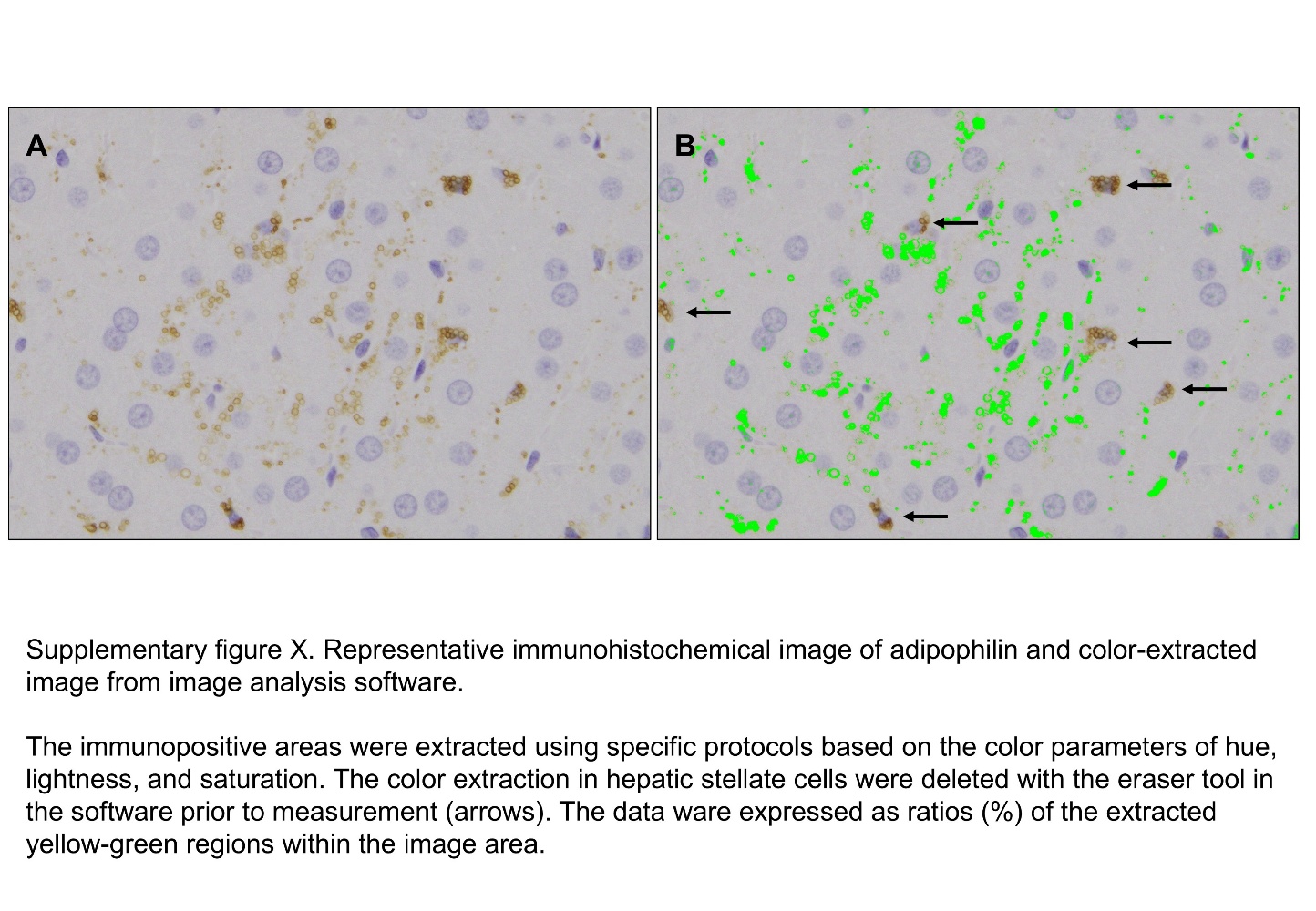
**

**Supplementary Figure S1:** Representative immunohistochemical image of adipophilin (A) and color-extracted image generated using image analysis software (B).

The immunopositive areas were extracted using specific protocols based on the color parameters of hue, lightness, and saturation. The immunopositive, adipophilin-expressing hepatic stellate cells (arrows) were deleted with a software eraser tool prior to measurement. The data are expressed as ratios (%) of the extracted yellow-green regions within the image area.


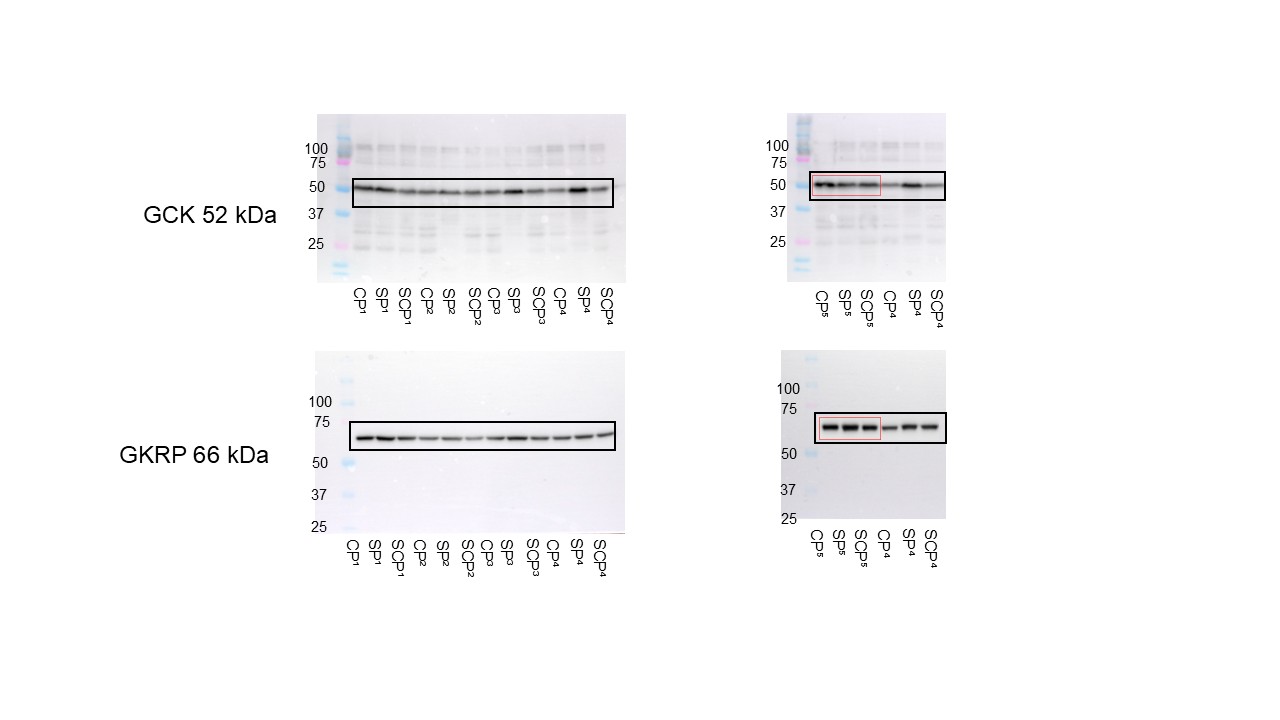


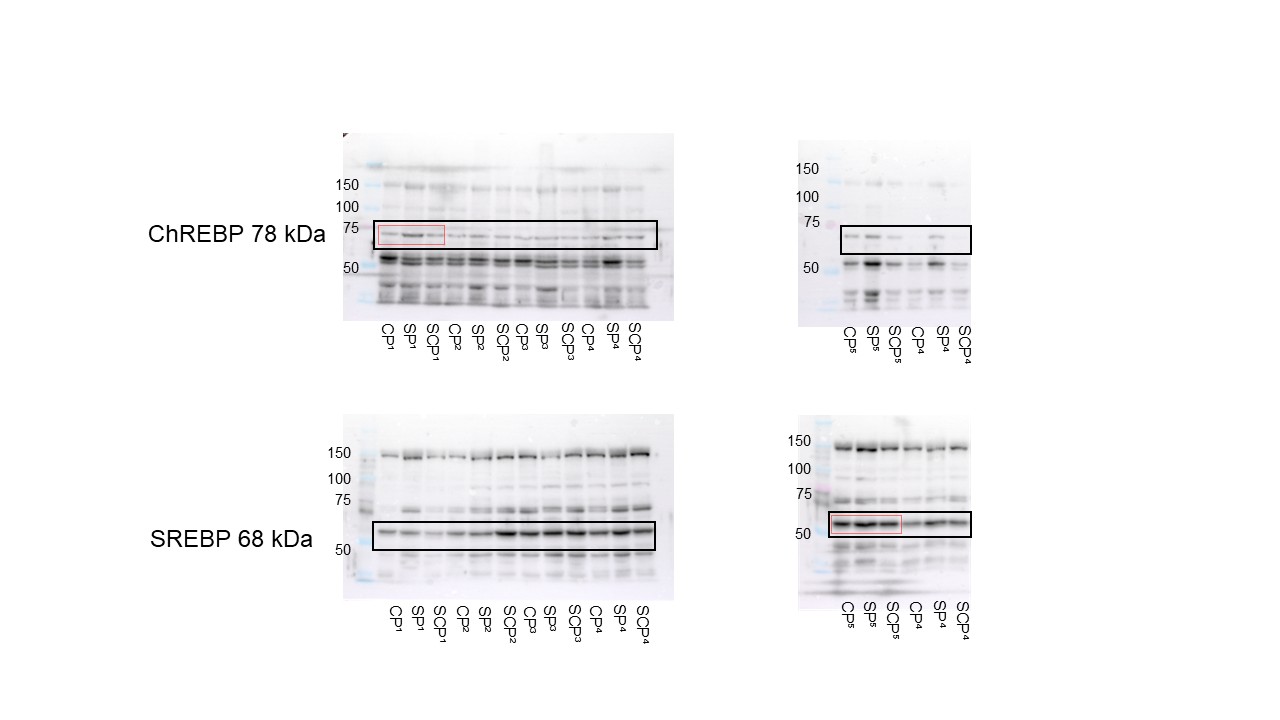
**
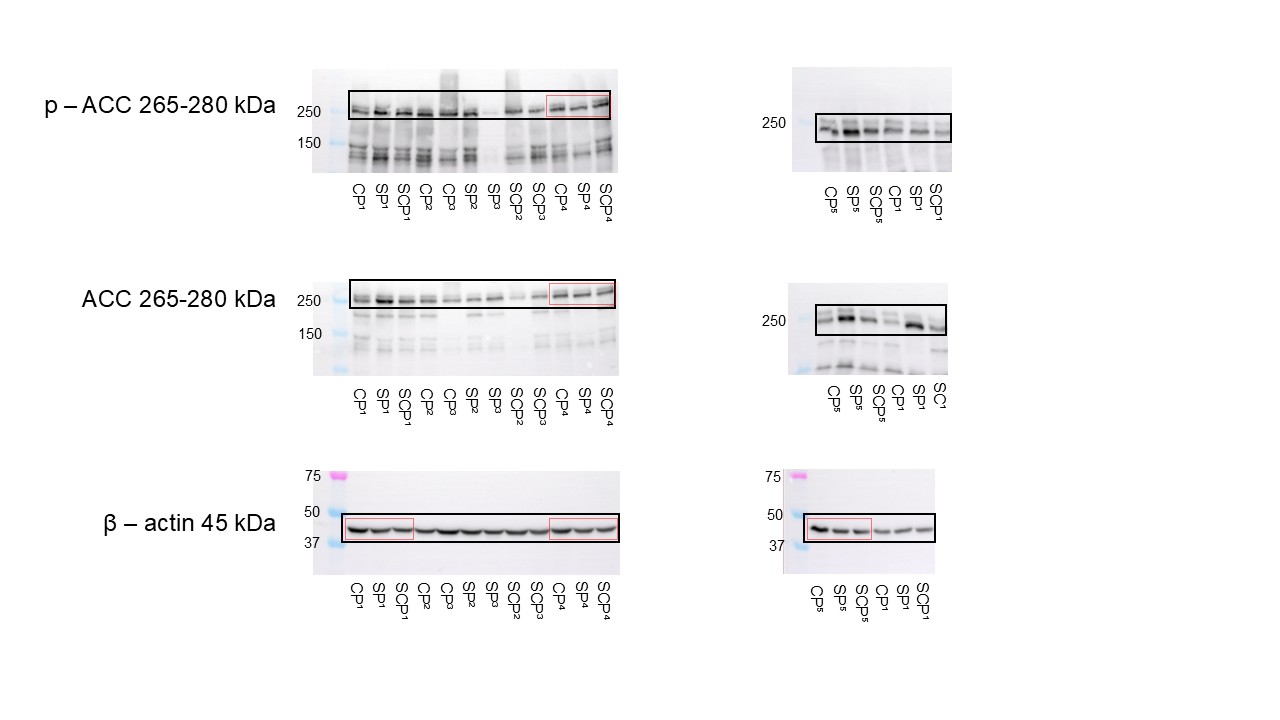
**

**Supplementary Figure S2:** Uncropped, full-length images of the western blot membranes presented in Figure 3H, 3I, 4A, 4B, 4C and 4D. The specific proteins of interest are indicated by a black frame. The cropped bands shown in the main figures are highlighted with a red frame.
